# The relationship between blood–brain barrier dysfunction and neurocognitive impairments in first-episode psychosis: findings from a retrospective chart analysis

**DOI:** 10.1192/bjo.2023.22

**Published:** 2023-04-11

**Authors:** Isabel Maurus, Sarah Wagner, Mattia Campana, Lukas Roell, Johanna Strauss, Piyumi Fernando, Susanne Muenz, Peter Eichhorn, Andrea Schmitt, Susanne Karch, Oliver Pogarell, Rolf R. Engel, Peter Falkai, Alkomiet Hasan, Elias Wagner

**Affiliations:** Department of Psychiatry and Psychotherapy, University Hospital, Ludwig Maximilian University of Munich, Germany; Department of Psychiatry, Psychotherapy and Psychosomatics, Medical Faculty, University of Augsburg, Germany; Institute of Laboratory Medicine, University Hospital, Ludwig Maximilian University of Munich, Germany; Department of Psychiatry and Psychotherapy, University Hospital, Ludwig Maximilian University of Munich, Germany; and Laboratory of Neuroscience (LIM27), Institute of Psychiatry, University of São Paulo, Brazil; Department of Psychiatry and Psychotherapy, University Hospital, Ludwig Maximilian University of Munich, Germany; and Max Planck Institute of Psychiatry, Germany

**Keywords:** Schizophrenia, cerebrospinal fluid, cognition, working memory, working speed

## Abstract

**Background:**

Even before the onset of psychotic symptoms, individuals with schizophrenia display cognitive impairments. Simultaneously, increasing amounts of individuals exhibit dysfunction of the blood–brain barrier (BBB). However, the impact of BBB dysfunction on neurocognitive impairment in people with first-episode psychosis has not yet been investigated.

**Aims:**

To advance understanding of said relationship, we considered one of the largest first-episode psychosis cohorts with cerebrospinal fluid parameters available, and investigated whether BBB dysfunction is related to working memory, working speed and attention.

**Method:**

We conducted a retrospective chart review of 121 in-patients diagnosed with a first episode of a schizophrenia spectrum disorder. Patients underwent neurocognitive testing and a lumbar puncture within routine clinical care. To define BBB dysfunction, albumin cerebrospinal fluid/serum quotients, immunoglobulin G ratios and oligoclonal band types were evaluated, and gender-specific differences investigated. Neurocognitive functioning was assessed by the Wechsler Adult Intelligence Scale, Test of Attentional Performance and Repeatable Battery for the Assessment of Neuropsychological Status. We performed simple and multiple linear regression analyses to interpret associations of interest.

**Results:**

Of those tested, 16% showed an alteration in albumin quotients and 12% had an oligoclonal band type indicating BBB dysfunction. Notably, male patients were more likely to have an increased albumin quotient and a higher immunoglobulin G ratio than female patients. We found no significant association between BBB dysfunction and neurocognitive assessments.

**Conclusions:**

The hypothesised relationship between BBB and neurocognitive impairments was not detectable in our retrospective cohort. Further cerebrospinal fluid-based studies with a longitudinal assessment of cognitive functioning and disease trajectory are urgently needed.

## Cognitive impairments in schizophrenia

The clinical manifestation of schizophrenia comprises considerably heterogeneous symptom patterns, aetiopathogeneses and disease courses. Cognitive impairments are one of the key characteristics of schizophrenia symptoms. They significantly explain variance in functional outcomes in those affected, and constitute one of the main limiting factors regarding recovery.^1^

Strikingly, cognitive impairments arise even before the prodromal phase and are a robust finding in people with first-episode psychosis (FEP).^2,3^ Although the impairments affect most cognitive domains, deficits in social cognition, working speed, working memory and verbal memory are the most pronounced in people with FEP.^3–5^ However, the extent of cognitive impairment varies considerably among individuals with FEP.^6^ Because of the major impact of cognitive impairments on functional outcome in schizophrenia, understanding the factors that account for those differences and their possible neurobiological underpinnings is crucial.

In general, both abnormal neurodevelopmental and neurodegenerative processes are presumed to be implicated in the aetiology of schizophrenia spectrum disorders, and might lead to either an impaired acquisition of cognitive abilities or their premature deterioration, respectively. Risk factors for schizophrenia spectrum disorders include maternal *in utero* infections, obstetric complications, and autoimmune and infectious neuroinflammatory processes. Remarkably, and of concern, is that all of these risk factors can result in blood–brain barrier (BBB) dysfunction.^7,8^

## BBB dysfunction and its clinical implications

The BBB is an endothelial diffusion barrier in the central nervous system (CNS) that restricts the permeability of potential pathogens and is essential for neuronal functioning.^9^ Correspondingly, chronic increased BBB permeability can lead to an accumulation of blood-derived neurotoxic proteins involved in neurodegeneration, disrupted glutamate homoeostasis and disturbed neural signaling.^9,10^ BBB breakdown is presumed to be an early event in the ageing human brain, beginning in the hippocampus and possibly contributing to cognitive impairment.^10^ Accordingly, BBB dysfunction has been suggested as an independent early biomarker of cognitive impairments in older adults.^11^ Moreover, increased exposure of CNS tissues to cytokines or antibodies can induce neuroinflammatory processes,^12^ which, in turn, are implicated in the aetiology of schizophrenia. Thus, BBB dysfunction may be of utmost importance regarding aetiopathological processes in schizophrenia spectrum disorders, thereby also potentially contributing to cognitive impairment.

## BBB dysfunction

To detect BBB dysfunction and quantify BBB permeability, cerebrospinal fluid (CSF)/serum albumin quotient is the gold standard in humans.^9^ Albumin concentrations are normally approximately 200 times lower in CSF compared with blood. Hence, elevated ratios indicate an increased passage of albumin (and other macromolecules) from blood to CSF as a result of BBB dysfunction.^9^ As previously established in a meta-analysis of case–control studies, albumin quotient levels are increased in people with schizophrenia.^13,14^ In comparison, only a handful of studies have explored indicators of BBB dysfunction in people with FEP. A previous investigation led by our group regarding people with FEP found an increased albumin quotient in about a sixth of patients.^15^ Another sample of 95 patients with FEP focused solely on total CSF protein levels and found said measures to be elevated in male patients.^16^

Additional markers can be used to study BBB dysfunction. The already cited meta-analysis of CSF markers concludes that individuals with schizophrenia display elevated immunoglobulin G (IgG) ratios when compared with healthy controls.^13^ Assuming an intact BBB, IgG levels in CSF are normally 400 times lower than in the serum.^9^ Increased IgG ratios are not only indicative of impaired BBB functioning, but also point to a higher intrathecal IgG production, possibly because of infectious or autoimmune inflammatory processes.^17^

Another possible marker of intrathecal immunoglobulin production, CNS inflammation and BBB dysfunction are CSF oligoclonal bands (OCBs). In the previously mentioned meta-analysis, abnormal OCBs were present in up to 12.5% of individuals with schizophrenia. In two previous FEP samples, OCBs indicating intrathecal IgG synthesis have been found in roughly 12% of cases, and an OCB pattern indicating BBB dysfunction was even found in 17% and 25%, respectively.^18,19^

To summarise, there are several CSF/serum markers for BBB functioning that are altered in people with schizophrenia spectrum disorders. However, to the best of our knowledge, the clinical implications of BBB dysfunction in people with FEP remain poorly understood and unestablished. The present study aimed to evaluate markers of BBB dysfunction in our sample. Furthermore, we investigated associations between said markers and neurocognitive performance with particular focus on key domains characteristically impaired in FEP. Our goal was to contribute to a growing body of knowledge in regard to the neurobiological underpinnings of neurocognitive impairments in people with schizophrenia spectrum disorders.

## Method

### Study population and data extraction

The authors assert that all procedures contributing to this work comply with the ethical standards of the relevant national and institutional committees on human experimentation and with the Helsinki Declaration of 1975, as revised in 2008. All procedures involving patients were approved by the local ethics committee of the Faculty of Medicine at the LMU Munich (registration number: 18-570). Informed consent was not needed (anonymously collected retrospective chart analysis of clinical routine data).

We conducted a retrospective chart analysis with clinical data from 166 in-patients with FEP admitted to our tertiary care hospital between 1 January 2008 and 1 August 2018 (Department of Psychiatry and Psychotherapy, University Hospital, Ludwig Maximilian University of Munich, Germany). Clinical data was extracted from patient records and the electronic clinical documentation system.

Inclusion criteria were that patients have a main diagnosis of F2x (schizophrenia spectrum according to the ICD-10) in the discharge summary of the first in-patient stay, and that patients had undergone both a lumbar puncture and neurocognitive testing within the clinical routine assessments before receiving this diagnosis. In our hospital, CSF analyses are usually offered to every FEP patient by the treating physicians so as to rule out organic psychoses, according to best clinical practice.

We also included patients who initially presented themselves with psychotic symptoms and a non-F2x-diagnosis (e.g. drug-induced psychosis), and were later diagnosed within the schizophrenia spectrum.

Exclusion criteria comprised any psychiatric or physical pre-diagnoses possibly resulting in cognitive impairments or BBB dysfunction (such as intellectual disability, perinatal asphyxia or stroke); abnormal brain magnetic resonance imaging findings suggestive of a relevant co-occurring CNS disorder (e.g. neoplasms); and a time interval of more than 21 days between CSF and blood sampling, or of more than 90 days between CSF sampling and neurocognitive testing, to ensure a clinically meaningful temporal association. Based on thorough screening of all available data, 45 patients were excluded before data analysis (see Supplementary Appendix 1). Thus, 121 patients were included in the final sample.

### CSF parameters

To investigate BBB dysfunction, albumin quotient, IgG ratio and OCB types were analysed. Additionally, the number of cells in the CSF was evaluated.

Based on two consensus report recommendations as detailed in a previous publication from our group,^15,20,21^ we distinguished five types of OCB patterns (type 1: normal CSF; type 2: two or more CSF-restricted OCBs; type 3: CSF-restricted OCBs plus identical OCBs in serum and CSF; type 4: identical OCBs in CSF and serum, also called ‘mirror pattern’; type 5: monoclonal bands in CSF and serum). Although OCB types 2 and 3 result from an intrathecal synthesis of IgG, OCB type 4, the ‘mirror pattern’, represents a systemic immune reaction and stems from passive transfer of OCBs from serum to CSF because of an increased BBB permeability.

### Neurocognitive parameters and interpretation

All neurocognitive tests were performed within routine care and diagnostic processes available to patients with FEP in our hospital. At the time of neuropsychological testing, patients were abstinent from drugs and in sufficiently stable condition to complete said testing procedures.

The present study focused on tests assessing working memory, working speed and attention. Working memory was assessed by means of the German version of the Wechsler Adult Intelligence Scale-III (Wechsler Intelligenztest für Erwachsene (WIE)),^22^ as well as the Test of Attentional Performance (TAP) version 2.1.^23^ The Repeatable Battery for the Assessment of Neuropsychological Status (RBANS^24^) was administered to measure attention. Because of clinic specific protocols and routine, it is important to note that neuropsychological testing procedures aimed to address cognition in a general as opposed to a manner specific to schizophrenia spectrum disorders. Nonetheless, they are established for this purpose.^25,26^

The WIE is a test battery to measure overall cognitive functioning. It comprises several subtests and allows to determine an index value for working memory. According to the definition of the WIE, working memory represents the individual's ability to process information. The three subtests of the WIE used to calculate the working memory index score consist entirely of verbal tasks. Working speed, as defined by the WIE, represents the individual's mental information processing speed. The raw values of two subtests of the WIE were used to calculate the working speed index score from the number of correct solutions (or the number of correct minus the number of incorrect solutions) within a certain time.

The TAP 2.1 is a digital assessment of different components of cognition including, but not limited to, working memory. It comprises 12 visual subtests with varying difficulty and complexity. For our purposes, raw scores of median reaction time in the working memory task were corrected for age, gender and education, and then converted to *T*-scores.

The RBANS is an instrument to measure impairments in different cognitive dimensions and consists of 12 subscales that allow to determine index scores for five cognitive domains and compare them to age-dependent norm values, with attention being one of them. Attention represents the individual's capacity to remember and modify information presented both visually and orally by means of short-term memory storage and modulation processes. More details on neuropsychological assessments can be found in Supplementary Appendix 2.

### Statistical analyses

All analyses were performed with IBM SPSS version 26.0.0.1 for Windows, with a significance level of *α* = 0.05. Before analyses, all non-dichotomous variables were *z*-standardised to ensure comparability between different scales, including age at the time of cognitive testing, educational level, albumin quotient, IgG ratio, OCB types and neurocognitive outcomes.

Gender-specific group differences between BBB parameters were compared with *t*-tests and *χ*^2^-tests. In case of a significant difference, the *ϕ*-coefficient was used to test the association strength of the variables.

First, we conducted simple linear regressions to determine whether BBB parameters were associated with working memory (WIE and TAP 2.1), working speed (WIE) and attention (RBANS). Second, we performed multiple linear regressions to control for the variables gender, age and educational level. Since TAP 2.1 raw scores must be corrected for age, gender and educational level before conversion to *T*-scores, we only used simple linear regressions to investigate possible associations with CSF parameters.

Additionally, we grouped individuals with any abnormal CSF findings (elevated age-dependent albumin quotient, OCB types 2–4, elevation of cells in the CSF) and compared said subgroup to the subgroup without abnormal values on all relevant neurocognitive domains, using *t*-tests.

## Results

### Demographic and clinical characteristics

In our final sample of *n* = 121, mean age at neurocognitive testing was 35.1 years (s.d. = 15.5), with 65 (53.7%) male and 56 (46.3%) female patients. Mean duration of illness at the time of lumbar puncture was 12.2 months (s.d. = 23.4). During their in-patient stay, 112 patients were treated with antipsychotic medication (92.5%) as recorded in discharge summaries, mostly with one antipsychotic agent (84 out of 121; 69.4%). More details on demographic and clinical characteristics of our sample can be found in Table 1.

**Table 1:** Demographic and clinical characteristics

Variables	*N* total	Frequency	
Gender (female/male)	121	56/65 (46.3%/53.7%)	
Diagnosis^a^	121		
Paranoid schizophrenia (ICD-10: F20.0)		49 (40.5%)	
Hebephrenic schizophrenia (ICD-10: F20.1)		03 (02.5%)	
Catatonic schizophrenia (ICD-10: F20.2)		02 (01.7%)	
Schizotypal disorder (ICD-10: F21)		01 (00.8%)	
Persistent delusional disorder (ICD-10: F22.X)		10 (08.3%)	
Acute and transient psychotic disorder (ICD-10: F23.0)		08 (06.6%)	
Acute polymorphic psychotic disorder with symptoms of schizophrenia and acute schizophrenia-like psychotic disorder (ICD-10: F23.1 and F23.2)		14 (11.6%)	
Other acute predominantly delusional psychotic disorders (ICD-10: F23.3)		04 (03.3%)	
Schizoaffective disorders (ICD-10: F25.0)		02 (01.7%)	
Schizoaffective disorder, depressive type (ICD-10: F25.1)		11 (09.1%)	
Schizoaffective disorder, mixed type (ICD-10: F25.2)		07 (05.8%)	
Others		10 (08.3%)	
Educational level	121		
High school diploma		51 (42.1%)	
Secondary school diploma		14 (11.6%)	
Middle school diploma		19 (15.7%)	
No degree (yet)		03 (02.5%)	
Not specified		34 (28.1%)	
Antipsychotic treatment^b^	121		
None		02 (01.7%)	
One		84 (69.4%)	
Two		27 (22.3%)	
Three		01 (00.8%)	
Not specified		07 (05.8%)	
Drug misuse before hospital admission^c^	121		
Yes, one substance		22 (18.2%)	
Yes, two substances		05 (04.1%)	
Yes, multiple substances		06 (05.0%)	
No		85 (70.2%)	
Not specified		03 (02.5%)	
		Mean	s.d.
Age at neurocognitive testing (years)	121	35.10	15.54
Females	56	35.07	15.94
Males	65	35.12	15.30
Premorbid IQ^d^	84	97.80	16.68
Duration of illness at the time of the lumbar puncture (months)	70	12.20	23.39

a. Diagnosis according to the ICD-10.

b. As recorded in discharge summaries.

c. Misuse of at least one substance (other than tobacco) before patients were admitted to hospital.

d. Assessed with the German version of the Wechsler Adult Intelligence Scale-III vocabulary task as described by Ayesa-Arriola et al,^27^ by using the formula proposed by Howard.^28^

### CSF and serum characteristics

An elevated age-dependent albumin quotient indicating BBB dysfunction was present in 19 out of 121 patients (15.7%). Type 4 OCBs were present in 14 out of 121 patients (11.6%). Mean albumin quotient was 5.4 (s.d. = 2.2) and mean IgG CSF/serum ratio was 2.8 (s.d. = 2.5). Three patients had an elevated number of cells in the CSF (≥5 per *μ*L, 2.5%). More details regarding CSF and serum characteristics are shown in Table 2.

**Table 2:** Cerebrospinal fluid and serum characteristics

Variables	*N* total	Mean	s.d.
CSF			
Albumin (g/L)	121	00.24	00.12
IgG (g/L)	121	00.03	00.02
Protein level (mg/dL)	119	36.53	12.94
Number of cells (per *μ*L)	121	1.40	1.25
		Frequency	
Elevated number of cells (≥5 per *μ*L)	121		
Yes		3 (2.5%)	
No		118 (97.5%)	
		Mean	s.d.
Serum			
Albumin (g/L)	120	44.83	4.00
IgG (g/L)	120	10.02	1.97
		Mean	s.d.
CSF/serum ratios			
Albumin quotient (CSF/serum)	120	5.35	2.22
IgG ratio (CSF/serum)	120	2.81	2.54
		Frequency	
Elevation age-dependent	121		
Yes		019 (15.7%)	
No		101 (83.5%)	
Not specified		001 (00.8%)	
OCBs^a^	121		
No OCBs/type 1		59 (48.8%)	
OCBs type 2		03 (02.5%)	
OCBs type 3		10 (08.3%)	
OCBs type 4		14 (11.6%)	
OCBs type 5		0 (0%)	
Not determined		35 (28.9%)	

CSF, cerebrospinal fluid; IgG, immunoglobulin G; OCB, oligoclonal band.

a. Five types of OCB patterns were proposed: type 1, normal CSF; type 2, two or more CSF-restricted OCBs; type 3, CSF-restricted OCBs and additional, identical OCBs in serum and CSF; type 4, identical OCBs in CSF and serum, ‘mirror pattern’; type 5, monoclonal bands in CSF and serum.

### No association between BBB dysfunction and working memory

Based on simple regression analyses (details can be found in Supplementary Appendix 3) and multiple linear regression analyses, no significant association between the WIE working memory score and albumin quotient (*ß* = −0.01, 95% CI −0.29 to 0.26, *P* = 0.920), IgG ratio (*ß* = −0.07, 95% CI −0.81 to 0.46, *P* = 0.586) or OCB types (*ß* = −0.19, 95% CI −0.48 to 0.06, *P* = 0.125) was observed. As indicated by the simple linear regression analyses, there was no significant relation between TAP 2.1 score and albumin quotient (*F*(1,80) = 1.46, *P* = 0.231), IgG ratio (*F*(1,80) = 1.76, *P* = 0.188) or OCB types (*F*(1,57) = 0.27, *P* = 0.607).

When we compared the subgroups of individuals with and without abnormal CSF findings, there were no significant differences between both groups (test statistics provided in Supplementary Appendix 3).

Hence, we found no significant association between BBB dysfunction and working memory in our data.

### No robust association between BBB dysfunction and working speed

Based on simple (details in Supplementary Appendix 3) and multiple linear regression analyses, no significant association between WIE working speed score and albumin quotient (*ß* = −0.07, 95% CI −0.34 to 0.20, *P* = 0.601) or IgG ratio (*ß* = −0.10, 95% CI −0.86 to 0.38, *P* = 0.436) was observed. As indicated by the simple linear regression analyses, there was no significant relation between WIE working speed score and OCB types (*F*(1,58) = 0.36, *P* = 0.549). However, when relevant covariates were included in the analysis, multiple linear regression showed a significant relation between the WIE working speed score and OCB types (*ß* = −0.25, 95% CI −0.54 to −0.01, *P* = 0.04). To determine whether there is a statistically significant difference in WIE working speed between the different OCB types, we additionally calculated a one-factor analysis of variance (ANOVA). The ANOVA did not show significant differences between the OCB types regarding WIE working speed (*F*(3, 56) = 0.56, *P* = 0.647). Considering the ANOVA and the relatively high *P*-value in the multiple linear regression analysis, we do not consider this finding to be robust. When we compared the subgroups of individuals with and without abnormal CSF findings regarding working speed, there were no significant differences between both groups (test statistics provided in Supplementary Appendix 3).

### No association between BBB dysfunction and attention

Based on simple (details in Supplementary Appendix 3) and multiple linear regression analyses, no significant association between the RBANS attention score and albumin quotient (*ß* = 0.01, 95% CI −0.26 to 0.27, *P* = 0.975), IgG ratio (*ß* = −0.05, 95% CI −0.72 to 0.52, *P* = 0.748) or OCB types (*ß* = −0.09, 95% CI −0.58 to 0.37, *P* = 0.656) was observed. Moreover, there was no significant difference between the subgroups of individuals with and without abnormal CSF finding.

Consequently, based on our analyses, we found no significant association between BBB disruption markers and attention. Full test statistics are provided in Supplementary Appendix 3.

### Comparison of albumin quotient elevation between female and male individuals

We found a statistically significant association between gender and age-dependent albumin quotient elevation (*χ*²(1) = 5.58, *P* = 0.018, *φ* = 0.22). Male patients were more likely to have an increased age-dependent albumin quotient than female patients. A coefficient of *φ* = 0.22 indicates a small effect. In addition, there was a statistically significant difference between IgG ratio in the male versus the female subsample (*t*(118) = 2.22, *P* = 0.028). Mean IgG ratio was 1.02 units (95% CI 0.11–1.92) higher in the male group (mean 3.28, s.d. = 3.30) than in the female group (mean 2.26, s.d. = 0.86). There was no statistically significant association between gender and OCB type detectable (*χ*²(3) = 1.93, *P* = 0.586). However, because three cells had an expected frequency smaller than five, interpretation of said results could be subject to error.

## Discussion

The present study examined the relationship between markers of BBB dysfunction and neurocognitive functioning in individuals with schizophrenia spectrum disorder and FEP. By doing so, we wanted to investigate whether biomarkers of BBB dysfunction might link pathogenetic factors such as neuroinflammatory processes to neurocognitive impairments in this population.

We found that 16% of patients showed an abnormal albumin quotient indicating BBB dysfunction; male patients were more likely to have an increased albumin quotient than female patients. Moreover, male patients had higher IgG ratios. In the total sample, 12% of individuals had type 4 OCBs. We found no significant association between said variables and neurocognitive domains assessed in our study.

To the best of our knowledge, the present study is the first to investigate and report on the relationship between BBB dysfunction and cognitive performance in people with FEP. Therefore, comparisons and interpretations related to the existing and relevant body of literature must be conducted carefully. Previous studies investigated the prevalence of BBB dysfunction and its implications for psychopathological symptom severity. For example, a cross-sectional study found an elevated albumin quotient and a lower IgG index in individuals with schizophrenia spectrum disorder when compared with healthy controls. Additionally, individuals in partial symptom remission had higher albumin quotients than those in full symptom remission.^29^

Another observational study included 95 FEP in-patients that underwent routine CSF diagnostics. The authors concluded that CSF glucose and lactate dehydrogenase were associated with symptom severity. However, said study comprised different CSF parameters, thereby complicating any meaningful comparison.^16^ An increased total protein concentration was determined in only 6% of patients.^16^

Notably, this sample also reported gender-specific differences in CSF-total protein concentration, thereby confirming our finding regarding albumin quotient and IgG ratios. This suggests that male patients with FEP might be more prone to BBB dysfunction, a finding demanding further investigation.

Endres et al evaluated CSF parameters from a sample of 992 patients (456 patients with schizophreniform and 536 with affective syndromes).^18^ Roughly 16% of patients with FEP (*N* = 188) had elevated albumin quotient, strikingly similar to our cohort. Overall, BBB dysfunction significantly correlated with more severe symptoms as assessed by means of the Clinical Global Impression Scale,^30^ more suicide attempts and higher clinical scores for disorientation, hallucinations and ego boundary disturbances. However, it must be stated that correlation coefficients were low.^18^

In conclusion, the previous literature indicates that BBB dysfunction can be found reliably in a considerable proportion of patients with FEP, and is even more frequent in male individuals. Moreover, this proportion seems to suffer from more pronounced symptoms, but effect sizes tend to be small. Presumably, divergent clinical presentations associated with markers of BBB dysfunction point to different aetiologic factors, such as neuroinflammatory processes. Although there is some evidence for this relationship regarding psychopathological symptoms, our study was intended to make a first contribution toward exploring a possible relationship concerning neurocognitive symptoms. Even if our analyses did not show an association between BBB dysfunction and the neurocognitive domains investigated, we are convinced that this relationship should be further addressed in the future. Eventually, biological markers as well as individualised treatment strategies might emerge from a better understanding of aetiological processes in schizophrenia spectrum disorders.

One of the strengths of our study is its large sample size compared with previous investigations, which is necessary to explore associations with presumably small effect sizes. Next, we utilised a thorough methodology to minimise the impact of potential confounders. The shorter duration of illness in our FEP sample when compared with multi-episode samples reduces antipsychotic exposure and possible neurodegenerative processes in the course of the disease. Moreover, we carefully considered covariates where available, and applied detailed inclusion and exclusion criteria.

However, our findings should be interpreted in the context of study limitations. First, we cannot rule out the presence of additional confounding factors that we failed to address. For example, previous drug misuse, symptom severity at the time of neurocognitive testing and psychotropic medications might have influenced the results of neurocognitive tests,^3^ and should be included as additional covariables in future investigations. Particularly, antipsychotics are considered to have anti-inflammatory properties^31^ and could have contributed to the absence of associations in our study.

Second, because of availability, we only investigated a small range of neurocognitive assessments even if their selection was based findings in previous literature. Future studies may use cognitive tests assessing other and/or further domains that may be more strongly associated with BBB dysfunction, such as social cognition. Additionally, schizophrenia-specific neuropsychological test batteries like the MATRICS Consensus Cognitive Battery should be used.^32^

Third, our study design was retrospective and does not include follow-up data. However, the latter may be helpful to survey associations between BBB dysfunction and cognitive impairments during the course of disease. Fourth, even if considered as the gold standard, the CSF parameters presently assessed only represent indirect markers of BBB dysfunction. Combining them with additional *in vivo* techniques to study BBB functioning (e.g. dynamic contrast-enhanced magnetic resonance imaging)^33^ may increase sensitivity for minimal BBB leakage conditions and might yield even more conclusive results. Fifth, the demographic and clinical characteristics of our sample might limit generalisability of our findings. Although an earlier age at onset in schizophrenia has been associated with an increased genetic susceptibility^34^ and a higher symptom burden,^35^ the average age in our sample was slightly higher than the typical age at onset in schizophrenia, potentially because treating physicians might have recommended CSF analyses more strongly to older patients with FEP.

Lastly, considering previous findings on associations between CSF variables and clinical symptoms, correlation coefficients tend to be small. Thus, our sample size might have been too small to detect significant associations, although our study contains one of the largest CSF FEP cohorts to date.

In conclusion, a substantial proportion of patients with schizophrenia spectrum disorders show altered CSF findings indicating BBB dysfunction. However, an association between CSF-based indicators of BBB dysfunction and impaired working memory, working speed and attention was not detectable in our retrospective cohort comprising individuals with FEP. Future CSF-based studies should employ standardised and longitudinal assessments of cognitive functioning and disease trajectory to further enlarge the body of knowledge regarding the neurobiological underpinnings of cognitive impairments in people with schizophrenia spectrum disorders.

## Data Availability

The data that support the findings of this study are available from the corresponding author, I.M., upon reasonable request.

## Supplementary material

For supplementary material accompanying this paper visit http://doi.org/0.1192/bjo.2023.22. click here to view supplementary material
