# Supplementary material for: The relationship between blood–brain barrier dysfunction and neurocognitive impairments in first-episode psychosis: findings from a retrospective chart analysis
Source: BJPsych Open. 2023 Apr 11;9(3):e60. doi: 10.1192/bjo.2023.22 (PMC10134348; doi:10.1192/bjo.2023.22)
Supplement: Supplementary file 1 [file S2056472423000224sup001.zip › bjp_S1_exclusions.docx]

**Supplement S1.** Detailed description of reasons for exclusion prior to data analysis.

| **Reason(s) for exclusion** | **n** | **Details** |
| --- | --- | --- |
| Psychiatric/ Psychological comorbidity | 8 | Intellectual disability 2x |
|  |  | Autism; intellectual disability |
|  |  | Suspected dementia |
|  |  | Suspected atypical autism |
|  |  | Differential diagnosis of alcohol hallucinosis |
|  |  | Attention deficit hyperactivity disorder 2x |
| Physical comorbidity | 11 | History of stroke 19 years ago |
|  |  | Palliative prostate carcinoma; skeletal metastases |
|  |  | History of left hemispheric ischemia; multimorbid patient |
|  |  | Suspected history of meningitis; developmental delay |
|  |  | Suspected epilepsy |
|  |  | Suspected history of inflammatory CNS disease |
|  |  | Epilepsy; post-traumatic brain lesion |
|  |  | XXYY chromosomal abnormality |
|  |  | Suspected Hashimoto's encephalopathy |
|  |  | Delayed development in infancy |
|  |  | Perinatal asphyxia |
| Abnormal MRI findings | 6 | Macroadenoma; global brain volume reduction |
|  |  | Suspected cortical dysplasia |
|  |  | Suspected history of pontine myelinolysis or stroke |
|  |  | Suspected glioma |
|  |  | History of cerebellar stroke  Suspected history of posterior inferior cerebellar artery stroke |
| Different main diagnosis | 5 | Differential diagnosis alcohol hallucinosis |
|  |  | Unspecified nonorganic psychosis; alcohol and cannabis addiction |
|  |  | Adjustment disorder, differential diagnosis prodrome |
|  |  | Suspected organic delusional disorder |
|  |  | Mental and behavioral disorder caused by cannabinoids (F12.5), differential diagnosis schizophrenia (F20) |
| Time interval between CSF & blood sampling > 21 days | 3 |  |
|  |  |  |
|  |  |  |
| Time interval between CSF sampling & cognitive testing > 90 days | 18 |  |
| Total n of excluded patients | 45 | (6 patients with more than one exclusion criteria fulfilled) |
