# Supplementary material for: The relationship between blood–brain barrier dysfunction and neurocognitive impairments in first-episode psychosis: findings from a retrospective chart analysis
Source: BJPsych Open. 2023 Apr 11;9(3):e60. doi: 10.1192/bjo.2023.22 (PMC10134348; doi:10.1192/bjo.2023.22)
Supplement: Supplementary file 1 [file S2056472423000224sup001.zip › bjp_S2_cognitive tests.docx]

**Supplement S2** Brief description of cognitive scores and subtests investigated

| **Test battery** | **Score** | **Name of the score specific subtests/value** | **Description of the score specific subtests/value** |
| --- | --- | --- | --- |
| WIE^1^ | Working memory index |  | Working memory index is a compilation of the subtests *letter number sequence*, *computational thinking* and *repeat numbers*. |
|  |  |  |  |
|  |  | Letter number sequence | The investigator reads a series of letters and numbers of different lengths to the subject. The subject has to memorize and then reproduce them, first repeating the numbers in ascending order and then the letters in alphabetical order. |
|  |  | Computational thinking | The investigator reads a series of arithmetic problems to the subject. The subject must solve them in his or her mind and answer orally. |
|  |  | Repeat numbers | The investigator presents a series of digit sequences of different lengths. The subject has to repeat the series verbally, partly in the same and partly in the opposite order. |
|  | Working speed  index |  | Working speed index is a compilation of the subtests *symbol search* and *complete images*. |
|  |  | Symbol search | In a limited time, the subject compares a group of abstract symbols with a target symbol and indicates whether the target symbol is in the search group. |
|  |  | Complete images | In a limited time, the subject looks at a series of images and has to name the essential part or detail that is missing from each image. |
| TAP 2.1^2^ | Working memory score |  | Working memory score consists of the working memory median. |
|  |  |  |  |
|  |  | Working memory median | A display in front of the subject presents a sequence of numbers. The subject has to react to certain simple or complex, mostly visual, in times (also) acoustic conditions by pressing one of two buttons as quickly as possible and without errors^3^. |

| RBANS^4^ | Attention index |  | Attention index is a compilation of the subtests *digit span* and *coding*. |
| --- | --- | --- | --- |
|  |  |  |  |
|  |  | Digit Span | The investigator reads a string of digits. The subject has to repeat the digits in the same order. The length of the digit string increases by one on each trial. |
|  |  | Coding | A page in front of the subject is filled with rows of boxes with a number from 1 to 9 above each box (in random sequence) and a blank space below the number. At the top of the page is a scheme with a unique, simple, geometric character beneath each of the numbers. Using the key, the subject must fill in the number corresponding to each character, for as many boxes as the subject can complete in 90 seconds. |

^1^Aster, M., Neubauer, A. & Horn, R. (Eds.) (2006). *Wechsler-Intelligenztest für Erwachsene WIE. Manual. Übersetzung und Adaptation der WAIS-III von David Wechsler*. Frankfurt am Main: Psychological Corporation.

2Zimmermann P. & Fimm B. (1995). *Testaufmerksamkeitsbatterie zur Aufmerksamkeitsprüfung (TAP)*. Herzogenrath: Psytest-Verlag.

3https://www.psytest.de/en/test-batteries/tap/subtests#working_memory, zuletzt aufgerufen am 15.06.2022

4Randolph, C. (1998). *Repeatable Battery for the Assessment of Neuropsychological Status (RBANS)*. San Antonio, TX: Psychological Corporation (Harcourt).
