# Supplementary material for: The relationship between blood–brain barrier dysfunction and neurocognitive impairments in first-episode psychosis: findings from a retrospective chart analysis
Source: BJPsych Open. 2023 Apr 11;9(3):e60. doi: 10.1192/bjo.2023.22 (PMC10134348; doi:10.1192/bjo.2023.22)
Supplement: Supplementary file 1 [file S2056472423000224sup001.zip › bjp_S3_test statistics.docx]

**Supplement S3.** Detailed test statistics and additional analyses.

**S3.1 Association between blood-brain barrier dysfunction and working memory**

Based on simple linear regression analyses, no significant association between the WIE working memory score and markers of BBB dysfunction was observed (AQ: (F(1,82) = 0.04, p = .843), IgG ratio: (F(1,82) = 0.00, p = .990), OCB types: (F(1,56) = 0.06, p = .811)).

The three multiple linear regression models with WIE working memory score as dependent variable and markers of BBB dysfunction, age, sex and educational level as predictors were significant (AQ: (F(4,62) = 5.96, R² = .28 (adjusted R² = .23), p < .001), IgG ratio: (F(4,62) = 6.06, R² = .28 (adjusted R² = .24), p < .001), OCB types: (F(4,43) = 6.08, R² = .36 (adjusted R² = .30), p = .001)).

There were no significant differences between the subgroups of individuals with and without abnormal CSF findings regarding WIE working memory scores, t(83) = 0.78, p = .440, as well as regarding TAP 2.1 working memory scores, t(81) = 0.73, p = .467.

**S3.2 Association between blood-brain barrier dysfunction and attention**

Based on simple linear regression analyses, no significant association between the RBANS attention score and markers of BBB dysfunction was observed (AQ: (F(1,67) = 0.00, p = .974), IgG ratio: (F(1,67) = 0.06, p = .813), OCB types: (F(1,47) = 0.97, p = .330)).

The three multiple linear regression models with RBANS attention score as dependent variable and markers of BBB dysfunction, age, sex and educational level as predictors were significant for (AQ: (F(4,41) = 3.26, R² = .24 (adjusted R² = .17), p = .021), IgG ratio: (F(4,41) = 3.30, R² = .24 (adjusted R² = .17), p = .020)) but not for (OCB types: (F(4,24) = 1.02, p = .419)).

There were no significant differences between the subgroups of individuals with and without abnormal CSF findings regarding RBANS attention scores, t(67) = 0.85, p = .401.

**S3.3 Association between blood-brain barrier dysfunction and working speed**

Based on simple linear regression analyses, no significant association between the WIE working speed score and markers of BBB dysfunction was observed (AQ: (F(1,84) = 0.06, p = .800), IgG ratio: (F(1,84) = 0.10, p = .755)).

The three multiple linear regression models with WIE working speed score as dependent variable and markers of BBB dysfunction, age, sex and educational level as predictors were significant (AQ: (F(4,63) = 6.56, R² = .29 (adjusted R² = .25), p < .001), IgG ratio: (F(4,63) = 6.68, R² = .30 (adjusted R² = .25), p < .001, OCB types: (F(4,44) = 6.28, R² = .36 (adjusted R² = .31), p < .001)).

There were no significant differences between the subgroups of individuals with and without abnormal CSF findings regarding WIE working speed scores, t(85) = 0.40, p = .690.

**S3.5 Additional analysis: No difference between high vs. low performers in WIE working memory score regarding AQ**

After median split of WIE working memory scores into low (values lower than group median) and high (values as high as group median or higher) performers, t-test showed no statistically significant difference between AQ of high vs. low performers, t(118) = 0.02, p = .983.
